# [Retracted] NR4A1-induced increase in the sensitivity of a human gastric cancer line to TNFα-mediated apoptosis is associated with the inhibition of JNK/Parkin-dependent mitophagy

**DOI:** 10.3892/ijo.2024.5666

**Published:** 2024-06-25

**Authors:** Hongzhu Yan, Feng Xiao, Jue Zou, Chengmin Qiu, Weiwei Sun, Minmin Gu, Li Zhang

Int J Oncol 52: 367-378, 2018; DOI: 10.3892/ijo.2017.4216

Following the publication of this paper, it was drawn to the Editor's attention by a concerned reader that certain of the data shown in Figs. 2A and 4F were strikingly similar to data appearing in different form in other articles written by different authors at different research institutes that were submitted to their respective journals at around the same time; moreover, the same data had apparently been included in the western blots featured in Fig. 5A to show the Parkin and mito-LCIII protein bands. As it was not clear what had been the original venue for the submission of the strikingly similar data here, the Editor requested that the authors send to us all the raw data underlying the affected figures; however, the authors were not able to comply with this request at the time of asking.

Given that the authors were unable to provide the supporting data as requested, the Editor of *International Journal of Oncology* has decided that this paper should be retracted from the Journal on account of a lack of confidence in the presented data. The authors were asked for an explanation to account for these concerns, but the Editorial Office did not receive a satisfactory reply. The Editor apologizes to the readership for any inconvenience caused.

